# Organotin(IV) Dithiocarbamate Complexes: Chemistry and Biological Activity

**DOI:** 10.3390/molecules23102571

**Published:** 2018-10-09

**Authors:** Jerry O. Adeyemi, Damian C. Onwudiwe

**Affiliations:** 1 Material Science Innovation and Modelling (MaSIM) Research Focus Area, Faculty of Natural and Agricultural Science, North-West University, Mafikeng Campus, Private Bag X2046, Mmabatho 2735, South Africa; Jerryadeyemi1st@gmail.com; 2 Department of Chemistry, Faculty of Natural and Agricultural Science, North-West University, Mafikeng Campus, Private Bag X2046, Mmabatho 2735, South Africa

**Keywords:** organotin(IV), dithiocarbamate, structure, biological properties, stereochemistry

## Abstract

Significant attention has been given to organotin(IV) dithiocabamate compounds in recent times. This is due to their ability to stabilize specific stereochemistry in their complexes, and their diverse application in agriculture, biology, catalysis and as single source precursors for tin sulfide nanoparticles. These complexes have good coordination chemistry, stability and diverse molecular structures which, thus, prompt their wide range of biological activities. Their unique stereo-electronic properties underline their relevance in the area of medicinal chemistry. Organotin(IV) dithiocabamate compounds owe their functionalities and usefulness to the individual properties of the organotin(IV) and the dithiocarbamate moieties present within the molecule. These individual properties create a synergy of action in the hybrid complex, prompting an enhanced biological activity. In this review, we discuss the chemistry of organotin(IV) dithiocarbamate complexes that accounts for their relevance in biology and medicine.

## 1. Introduction

Metals are relevant for medicinal applications because they play crucial roles in the living systems of organisms. They become soluble in biological systems by losing electrons to form positively charged species. It is in this ionic state that they play their biological roles [1]. Metal ions lack electrons, while biological molecules like DNA and proteins are electron-rich, thus encouraging the propensity for these opposing charges of both metal ions and biological molecules to interact and bind. This tendency is also applicable to the interaction of metal ions with other small molecules and ions that are crucial to life, such as oxygen [1]. Tin, a group V metal, can be found in numerous inorganic/organometallic compounds. It has two 5s and two 5p electrons in its outer shell. It is able to lose the two electrons in the 5p orbital to form Sn^2+^ ions, or share all four electrons with other atoms by acquiring the stable electronic configuration of xenon. The chemistry of tin, mostly revealed in aqueous media, is more convoluted, because it uses inert electron pairs (a result of poor shielding of the outer electrons compared to those in the inner core) to direct its stereochemistry. Neither Sn^4+^ nor Sn^2+^ is found in aqueous solutions. Tin can bond with carbon to form a group of compounds known as organotin compounds [2]. Organotin compounds have contributed immensely to the study and the understanding of organometallic compounds, which began in 1949, and this has led to their usage in diverse application fields. Detailed studies on the structural properties and changes which occur in solution and solid states of organotin compounds have been reported [3].

Organotin compounds have at least one covalent carbon–tin bond, and are mostly tetravalent in structure [4]. These compounds were first discovered by Edward Frankland in 1894, with the first compound being diethyltindiiodide ((C_2_H_5_)_2_SnI_2_). Since two oxidation numbers are known for tin (+2 and +4), organotin compounds can exist as organotin(II) (sp^2^ hybridized) or organotin(IV) (sp^3^ hybridized) species. However, the +IV oxidation state has been found to be more stable than their +II state which oxidizes easily to their +IV state and often polymerizes. The only known stable organotin(II) compound is bis(cyclopentadienyl)tin(II) [3], but many organotin(IV) compounds are known and they conform to the general formula R_x_Sn(L)_4-x_, where R = alkyl or aryl substituents; L = organic or inorganic ligand [5].,

The presence of one or more covalent C-Sn bonds affects the activity of the whole compound depending on the number and nature of the alkyl (R) substituents attached to the Sn center [6]. Variation of the alkyl or aryl substituent in the organotin(IV) compounds has shown notable effects on the biological activities of these compounds [7]. These compounds contain a Sn centre with an anion, usually oxide, hydroxide, chloride, fluoride, carboxylate or thiolate [7]. They are widely used organometallic compounds and have been used over the last few decades for different applications in industry and agriculture such as pesticides, fungicides and as anti-fouling agents [8]. Organotin(IV) compounds have unique characteristics such as catalytic and redox capacity, structural diversity, the tendency for ligand exchange and a variety of available interactions with biologically useful properties [9]. Derivatives of organotin(IV) compounds have shown great therapeutic potential on diverse tumor cells, although their specific mode of action still remains unclear [10]. They have been reported to show good biological activities as an antimicrobial, schizonticidal, antitumor, antimalarial and as biocides in agriculture [11,12,13,14]. Their cations can easily form complexes with ligands possessing S, N, O and P donor atoms with various composition and stability [7]. An example of such ligand is the dithiocarbamate group.

Organotin(IV) dithiocabamate complexes have received attention due to their ability to stabilize specific stereochemistry in their complexes, and their diverse application in the field of agriculture, biology, catalysis [15] and their usefulness as single source precursors for tin sulfide nanoparticles are notable reasons for their modern relevance [16]. These complexes possess unique stereo-electronic properties which underline their relevance in the area of medicinal chemistry [17,18,19,20]. Organotin(IV) dithiocarbamates, therefore, owe their functionalities and usefulness to the individual attributes of the organotin and the dithiocarbamate moieties present within the molecule. For example, due to the presence of two sulfur atoms in the molecule, dithiocarbamates ligands show strong metal binding capacity, and this has encouraged a growing interest in this field of sulfur donor ligands [21]. This is the reason for their proven capacity to inhibit enzymes and ultimately affect biological environments [22]. This property has encouraged a growing interest in their usage as anticancer, antibacterial, antifungal and several industrial applications such as in rubber vulcanization [23,24]. Thus, the synergy exhibited by the organotin(IV) moiety and dithiocarbamate ligands has resulted in the enhanced biological activity of this hybrid molecule and this has led to the large interests in the chemistry and biological relevance of these complexes. Dithiocarbamate (DTC) ligands are very useful in complex formation [25] due to the possible functionalization of the substituents on the nitrogen atom of the dithiocarbamate backbone, which helps create various analogues of this compound. This has resulted in varieties of structures with refined attributes, which, consequently, enhances physical and chemical properties [26]. In this survey, some of the applications of organotin(IV) dithiocarbamates as biologically useful compounds will be reviewed. This is not intended to be exhaustive, but designed to discuss the chemistry and also highlight recent and exciting advances of this group of organometallic complexes in the area of medicine and biology.

## 2. Chemistry of Organotin(IV) Dithiocarbamate

**Figure 1:**
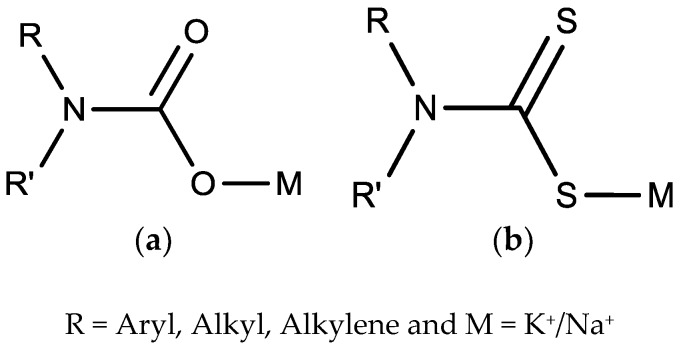
Structure of carbamate (**a**) and dithiocarbamate (**b**).

Dithiocarbamates are the hemi-amides of dithiocabonic acids [27]. They belong to the carbamate family in which the two oxygen atoms have been replaced with sulfur atoms (Figure 1). They are mono-anionic chelating ligands, and are capable of forming stable complexes with a large number of the main group elements, all transition metals and also lanthanides and actinides [28]. This is because of the presence of the anionic CS_2_¯ moiety which has a wide range of binding modes, thus, possessing good coordination capacity. This interesting coordination and stability observed between dithiocarbamate ligands and a metal derivative like organotin salts is based on the fact that some metals (like tin) act as strong Lewis acids [29,30], thus, they easily complex with the electron rich sulfur dithiocarbamate ligand based on the Hard Soft [Lewis] Acid Base (HSAB) principle [31]. They show good solubility in water or organic solvents depending on the nature of the cation.

**Figure 2:**
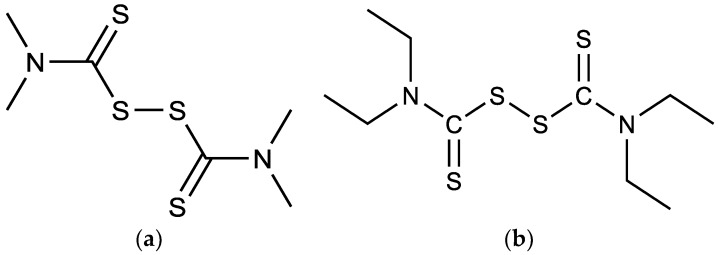
Structures of (**a**) thiram and (**b**) disulfiram.

Various biological activities can be prompted when incorporated into other molecular structures. Thus, they are good pharmacophore and have been shown to possess useful biological activities such as their usage as antifungal drugs. Thiram (Figure 2a): for food dressing and control of turf diseases and disulfiram (Figure 2b): for the treatment of addiction to alcoholic drinks [32,33]. However, the original and still the main use of disulfiram is as a catalyst in rubber vulcanization [34].

Dithiocarbamate compounds are capable of inhibiting the growth of bacteria by altering the metabolic activities which take place in the bacteria. This ligand continues to receive increased attention due to the presence of the carbon-sulfur bond which is useful in many products of biological and medicinal relevance [35]. This bond is also useful in the formation of organic intermediates with interesting chemistry [35,36]. Reports on their usage in protein folding, redox signaling and enzyme catalysis have been attributed to the strong nucleophilic character and peculiar properties of sulfur to undergo redox reaction [33]. They have found other usage in agriculture as pesticides, herbicides, and fungicides [37,38,39]. Some compounds of these classes have been commercialized such as zineb, maneb, nabam, ziram and ferbam [40].

**Scheme 1:**
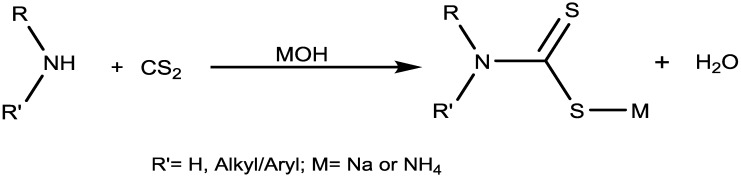
Preparation of dithiocarbamate from primary and secondary amine.

The earliest synthetic record of dithiocarbamate synthesis involved the use of thiophosgene, chlorothioformate and an isothiocyanate [40]. This had many drawbacks such as long reaction time, use of expensive and toxic reagents, and harsh reaction conditions. Several synthetic procedures have been developed involving one-pot condensation of amines, carbon disulfide and electrophiles such as alkyl halides, epoxides, carbonyl compounds and α,β-unsaturated compounds [40]. They have been prepared from both the primary and secondary amine [32,41,42] as shown in Scheme 1. The respective amines react with carbon disulfide in the a strong base like potassium/sodium hydroxide or ammonium hydroxide to form the corresponding dithiocarbamate salts of sodium ion (K^+^/Na^+^) or ammonium ion (NH_4_^+^). In this reaction, the addition of the strong base is to catalyze the reaction, hence making an important impact on the dithiocarbamate formation rate [32]. The dithiocarbamates obtained from secondary amines are more stable than the products obtained from primary amines due to the presence of acidic hydrogen on the nitrogen [32]. The Na^+^ or NH_4_^+^ salts of DTCs are reasonably stable compared to their corresponding carbamic acids. The sodium salts of DTCs are white crystalline solids which are soluble in water. These salts are usually stable for a long time, nevertheless whenever required; the solutions of DTCs are to be prepared freshly.

Another reported method for the synthesis of dithiocarbamates which are soluble in organic solvents involves the reaction of two equivalents of a secondary amine (in the absence of any base) with carbon disulfide. One equivalent of amine acts as the base while the other as a nucleophile for the reaction to give the ammonium salt of the compound [43].

Several metal dithiocarbamate complexes have been synthesized simply by the reaction of dithiocarbamate ligand with the corresponding metal salts in an appropriate solvent [43,44,45,46,47,48,49]. The metal dithiocarbamate complexes formed are often via simple metathesis with corresponding metal salt [15,50]. Some metal complexes of dithiocarbamate are colored, and this has been useful in facilitating their spectrophotometric studies especially in visible or near UV region. Most heavy metal dithiocarbamate compounds are sparingly soluble in solvents that are organic in nature such as chloroform, alcohols, dimethyl sulfoxide and dimethyl formamide [51,52].

**Figure 3:**
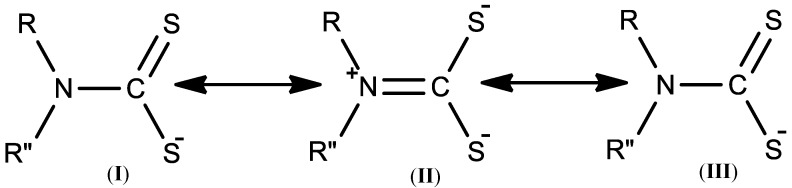
Resonance structure of dithiocarbamate.

With an appropriate choice of the substituents on the secondary amine, and a right choice of cation, a unique modification in the stereo-electronic properties of the corresponding dithiocarbamate ligand can be obtained [17]. Dithiocarbamates are lipophilic and possess the ability to bind to metal ions as monodentate (**I**) (using one binding site of S), as bidentate (**II**) (using the two S atoms in binding to the central metal atom or ion) or bidentate bridging ligands (**III**) (in which it binds to the metal ion with one sulfur and forms a bridge with adjacent molecule with the other sulfur) [53]. This is because DTC can exist in 3 different resonant forms [54] as shown in Figure 3. In the resonance form of the dithiocarbamate, there is a single bond between the nitrogen atom and the C bearing the two S atoms as presented in (**I**) and (**III**), and the delocalization of the -1 charge between the carbon and two sulfur atoms. The lone pair on the nitrogen atom (N) in the ‘thioureide’ form is delocalized as shown in the form (**II**), resulting in π (double bond) character between the N and the C bearing the two S groups with negative charges. The nitrogen is sp^2^ hybridized in this resonance form. The resonance form (**II**) is described as a hard ligand because it can stabilize hard metal centers at higher oxidation states while both forms (**I**) and (**III**) can stabilize soft metals at lower oxidation states [43].

The exceptional stability of dithiocarbamate is often explained by the outstanding contribution of resonance form (**II**) to the overall electronic structure, which ensures that this anion is an effective ligand for metal complexation. They exhibit various modes of coordination in homo and heteronuclear complexes, depending on the mode of attachment between the ligands and the metal ion [37]. The ease of replacement of hydrogen from -SH group in dithiocarbamate and their complexes in inorganic analysis is the main reason for their numerous applications. This usually occurs when a coordinate bond is formed through S, forming strongly colored complexes with a number of metals. This outstanding metal binding capacity of the dithiocarbamates was noted by Delepine [55].

The interesting chemistry of organotin(IV) dithiocarbamates results from the individual properties of both the organotin and dithiocarbamate moieties. As such, the chemistry and the application of these sets of complexes is believed to be the consequence of the synergistic activity of both the organotin and dithiocarbamate compound. Generally, different methods have been used to prepare organotin(IV) complexes of dithiocarbamate, and there is no specific method of synthesis. However, organotin(IV) dithiocarbamate complexes have been mostly reported to be prepared in situ. The in situ method involves a one pot system in which the organotin(IV) compound is introduced into the flask containing freshly prepared dithiocarbamate, and allowed to react for a few hours [40]. Some organotin(IV) dithiocarbamate complexes prepared via this method have been reported. Bashira et al., synthesized organotin(IV) dithiocarbamate complexes using “in situ” insertion method, involving the reactions of bis-2-methoxyethyl dithiocarbamate with some organotin compounds of the type R_x_SnCl_x_ (where R = Bu, Me, Ph and x = 1, 2 or 3) [56]. Organotin compounds with the molecular formula R_m_Sn[S_2_CN(CH_3_)(C_6_H_11_)]_4-m_ (where m = 2, R = CH_3_, C_2_H_5_; m = 3, R = C_6_H_5_) were reported by Awang et al., [24]. They also prepared organotin(IV) dithiocarbamate complexes derived from methoxyethyldithiocarbamate, with corresponding di- and tri-organotin chloride bearing alkyl group (R_3_SnL and R_2_SnL_2_;L = R = C_6_H_5_ or C_4_H_9_) [57]. Kamaludin et al., reported some organotin(IV) *N*-butyl-*N*-phenyldithiocarbamate compounds using the dithiocarbamate derivatives obtained from *N*-butylamine and the corresponding organotin chloride in different ratios [58]. The number of anions (e.g., Cl^−^) present in the organotin(IV) salt often determines the mole ratio of the dithiocabamate ligand to the organotin(IV) salts, because the chloride ion are labile and are easily replaced by the dithiocarbamate ligand. Thus, the molar ratio of the dithiocarbamate ligand to the organotin salt (for a substituted derivative) is 2:1 for di-substituted organotin(IV) salt and 3:1 for a tri-substituted derivatives.

Various spectroscopic techniques have been used to characterize organotin compounds and they give insight on the geometry of the compound. In the infrared spectroscopy, specific bands, aid in identifying the dithiocarbamate. Characteristic bands such as the thioureide band υ(C=N), the υ(C-S) and υ(M-S) band are peculiar. The thiouride band is often found in the 1450–1550 cm^−1^ band region of the spectrum, which is associated with the vibration of C-N bond that displays a partial double bond and a polar character. The υ(C-S) stretching vibrational band often appears in the 950–1000 cm^−1^ region, while the υ(M-S) stretching vibration band is found in the 350–450 cm^−1^ [59]. The movement of the C-N vibrational band after complexation with a metal to a higher frequency of about 15 cm^−1^ often indicates the delocalization of the electron cloud of the –NCSS group towards the metal center [60]. Thus, giving the C-N bond a partial double bond character [61]. The υ(M-S) band usually appears in the far infrared region, and indicates that complexation occurred [62]. In dithiocarbamate ligands, there are often two types of υ(C-S) band, the υ(CS_2_)asym and υ(CS_2_)sym; and they appear around 1055 cm^−1^ and 961 cm^−1^ respectively [63]. When these are replaced by strong singlet at approximately 1000 cm^−1^ in complexes, it indicates a symmetrical coordination of the dithiocarbamate moiety to the metal ions. However, the splitting of the same band within a difference of 20 cm^−1^ in the same region is ascribed to the monodentate binding mode of dithiocarbamate ligand [64].

The electronic spectra of organotin(IV) dithiocarbamate complexes are generally characterized by three principal bands which are attributed to (C=N) bond, the electron pair of sulfur and the metal-ligand (M-L) bond in the UV-visible absorption spectra [65]. From the dithiocarbamate moiety, the absorption band of (C=N) chromophore due to the intramolecular π-π* transition is found around 300 nm [56]. However, the movements of this peak to a lower/shorter wave length often reveals the involvement of the band in complex formation. This, therefore, shows the contribution of the NCS_2_ group in the complexation of the dithiocarbamate ligand to the organotin(IV) moiety [65]. The existence of a nonbonding electron pair of the S atom in the range 240–261 nm has been attributed to n-π* transition [65]. Furthermore, the presence of a broad shoulder band in the complexes, which is usually found above 300 nm, is attributed to charge transfer transition from the metal to the ligand (M-L). The absorption indicates an extended conjugation system due to the electronic transition between p-orbital of sulfur and 5d-orbital of tin metal [56,65,66].

In the characterization of organotin(IV) complexes, one of the widely and often used techniques useful for the prediction of geometry in organotin(IV) complexes is NMR spectroscopy. Parameters such as the coupling constant (*J*) [*^n^J*(^119^Sn,^1^H), *^n^J*(^119^Sn,^13^C)], and the chemical shifts (δ) of ^119^Sn-NMR are obtained from this technique and these give useful information in solution state about the geometry of the tin center in the organotin(IV) complexes [67]. Studies have shown that the coordination of tri and dimethyl derivatives of tin(IV) compounds having *^n^J* (*n* = 1, 2) values are: tetra-coordinated in tin(IV) compounds, if the coupling constant (*^1^J*) are predicted to be less than 400Hz then *^2^J* values should be less than 59 Hz; penta-coordinated, if the coupling constant (*^1^J*) is between 450 and 670 Hz and *^2^J* values is between 65 and 80 Hz; and hexa-coordinated, if the coupling constant (*^1^J*) is greater than 670 Hz and *^2^J* values is greater than 83 Hz [67,68]. These observed *J* values could be utilized in the calculation of the bond angle (θ) of C–Sn–C in solution using Lockhart’s equation, and the equation for the phenyl and the butyltin(IV) compounds were proposed by Howard et al. [69]:

θ = 0.0105 |*^2^J*(^119^Sn–^1^H)|^2^ − 0.799 |*^2^J*(^119^Sn–^1^H)| + 122.4 (1)

|*^1^J*(^119^Sn–^13^C)| = 11.4 θ − 875 (2)

(for methyl and ethyl derivatives).

|*^1^J*(^119^Sn–^13^C)| = (9.99 ± 0.73)θ − (746 ± 100) (3)

(for butyl derivatives).

|*^1^J*(^119^Sn–^13^C)| = (15.56 ± 0.84)θ − (1160 ± 101) (4)

(for phenyl derivatives).

These bond angles (θ) are attributed to tetracoordinated geometry, if θ ≤ 112°; penta-coordinated, if θ = 115–130°; and hexa-coordinated, if θ = 129–76° [67]. The tin chemical shift (^119^Sn) values often indicate the coordination number around the tin center and, thus, provide valuable information about the geometry of organotin(IV) complexes [67]. The shifts are, however, found to be dependent on the nature of the group attached to Sn. Caution is important in this analysis because tin resonance is strongly dependent on factors such as temperature, concentration used and electronegativity of the ligands [67,70,71]. With an electron donating group, Sn atom becomes more shielded, thereby leading to an increase in the chemical shift value [67]. The spectra of ^119^Sn-NMR of an organotin(IV) complex usually show a singlet which is significantly lower in frequency than corresponding organotin(IV) salts [71]. The lower chemical shifts observed in the spectra of organotin(IV) complexes are due to the change in the coordination number and bond angle around the tin center, caused by possible presence of electronegative substituents and dπ–pπ bonding effect. Hence, lower frequency upon coordination is an indication of increased coordination number [72]. The ^119^Sn-NMR shifts for organotin(IV) complexes are either tetra-(δ = 200 to −60 ppm), penta-( δ = −90 to −190 ppm) or hexa (δ = −210 to −400 ppm)-coordinated [73]. Chemical shifts (δ) in the range of −600 to −700nm suggest either 6- or 7-coordination geometry around the tin center. This has been attributed to a possible chemical equilibrium between octahedral and pentagonal-bypiramidal arrangement due to intramolecular metal ligand bond [74].

X-ray single crystal technique, on the other hand, exploits the fact that X-rays (in the Ångström range, ~10^−8^ cm) are scattered (diffracted) by electron cloud of an atom with similar sizes to determine the three dimensional structures of molecules [75]. Accurate knowledge of molecular structures are known to aid the development of effective drugs [76] and therapeutic complexes. This is achieved by the growth of crystal, which when diffracted gives useful information such as bond distances, bond angles, and can clearly show the geometry of molecules. This analytical technique gives clearer information compared to spectroscopic techniques which are often limited. However, X-ray single crystal diffraction analysis is limited since the molecule is in the solid state and not much information is obtained about the behavior of the molecule in solution [77].

Generally, the structural geometries of tin(IV) complexes are tetrahedral, trigonal pyramidal, or octahedral. For organotin(IV) compounds, the tri-substituted (R_3_SnX) and the di-substituted (R_2_SnX_2_) derivatives often possess five and six coordinate geometries respectively [2,78]. Thus, the high coordination number often observed in organotin(IV) complexes of the formula R_n_SnX_4–n_ (n = 1–3, X = halide, carboxylate, dithiocarbamate etc.) with Lewis bases is due to the increase in the Lewis acid strength of the tin metal [3]. These geometries are often influenced by the mole ratio of the ligand to organotin(IV) salt used. These coordination geometries have been found to depend on the mode of bonding in the dithiocarbamate moiety, which is mostly monodentate or bidentate fashion [79].

**Figure 4:**
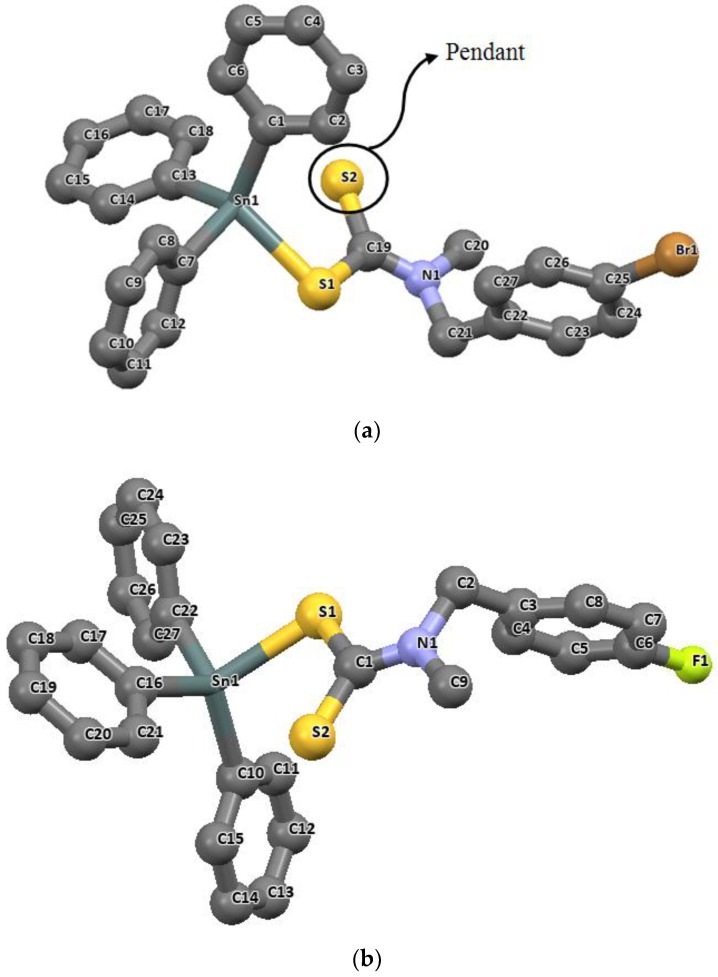
Molecular structures of (**a**) triphenyltin(IV) *p*-bromo-*N*-methylbenzylamine dithiocarbamate and (**b**) triphenyltin(IV) *N*-methylbenzylaminedithiocarbamate with ellipsoidal displacement at 50% probability level (For clarity, H atoms were omitted). Redrawn from [80], with permission from Elsevier (Copyright 2018).

Four coordinate geometries are mostly common for the tri-substituted organotin(IV) complexes. These complexes are not completely tetrahedral but are distorted due to the distance of the second non-coordinated sulfur atom which often hangs as a “pendant” as shown in Figure 4. The structures of triphenyltin(IV) complexes of *p*-bromo-*N*-methylbenzylamine dithiocarbamate and *p*-fluoro-*N*-methylbenzylaminedithiocarbamate have been reported [80]. Both complexes contain discrete molecular units with the central tin atom bonded to the triphenyltin moeties in a monodentate fashion, forming a supposed tetra-coordinated geometry as presented in Figure 4a,b. The bond lengths and angles observed for Sn-S bonds show that in both complexes, the modentate bond was through one of the sulfur atoms with the distance of 2.464(10) Å and 2.4671(4) Å. The bond lengths of the non-coordinating sulfur atoms with the tin metal was found below the van der Waal radii of 4.0 Å, but too weak because of the wide distances between Sn1-S2 bonds (3.022(7) Å and 3.066(8) Å). The bond angles S1-Sn1-C7 and C1-Sn1-C13 in triphenyltin(IV) *p*-bromo-*N*-methylbenzylaminedithiocarbamate are 90.54(9)° and 114.62(13)°; and in triphenyltin(IV)*N*-methylbenzylaminedithiocarbamate, the S1-Sn1-C13 and C1-Sn1-C7 are 92.82(2)° and 119.75(14)° respectively, which showed a deviation from the expected 109.5°. The wider angles show deviation from the expected tetrahedral angle, and have been attributed to the influence of the proximity of the non-coordinated sulfur atoms in the complexes [80].

**Figure 5:**
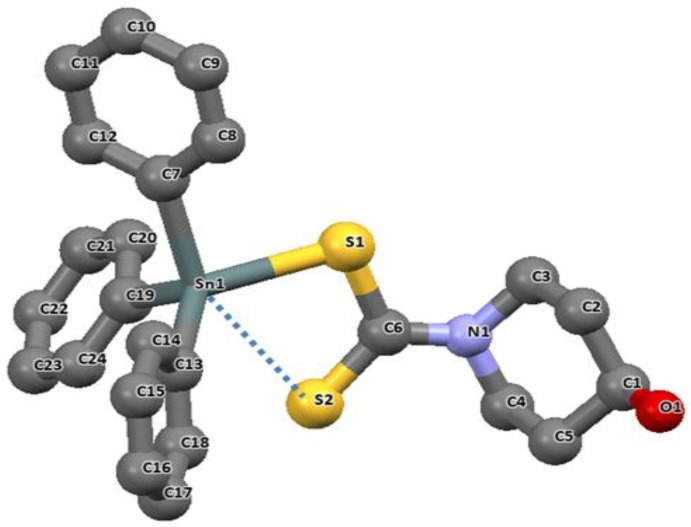
The molecular structure of triphenyltin(IV) 4-hydroxypiperidine dithiocarbamate. (For clarity, H atoms were omitted). Redrawn from [81], with permission from Elsevier (Copyright 2018).

Yandav et al., reported a monodentate coordination for the organotin(IV) complex obtained from 4-hydroxypiperidine dithiocarbamate. The ligand imposes a distorted tetrahedral geometry around the tin center as shown in Figure 5 [81]. The covalent Sn–S bond distances are unequal with Sn1–S1 and Sn1–S2, having distances of 2.4757(7) Å and 3.0336(7) Å respectively. The longer distance (3.0336(7) Å) is significantly high, but less than the sum of the van der Waal radii of two atoms, which suggested a weak interaction for the Sn1–S2 bond. Thus, the geometry of the coordination around the tin atom suggests a deviation away from the regular tetrahedron to a trigonal bipyramidal structure due to the weak bond interaction of Sn1–S2. This complex has subtending angles for the atoms in the axial and equatorial positions smaller than the ideal tetrahedral, which supports the deviation towards trigonal bipyramidal (for equatorial substituents C19–Sn1–C13 = 118.12(10)°; S1–Sn1–C19 = 112.75(7)°; S1–Sn1–C13 = 116.54(7)°).

Similarly, the structures of triphenyltin(IV) *N*-cyclohexyl-*N*-ethyl- and *N*-cyclohexyl-*N*-methyldithiocarbamate were reported to have anisobidentate coordination with one Sn-S short distance Sn1–S2 = 2.9426(10) Å and another long Sn1–S2 = 3.0134(8) Å, resulting in a distortion in geometry. Thus, the geometry in both complexes about the Sn center is between tetrahedral and trigonal bypiramidal [82].

**Figure 6:**
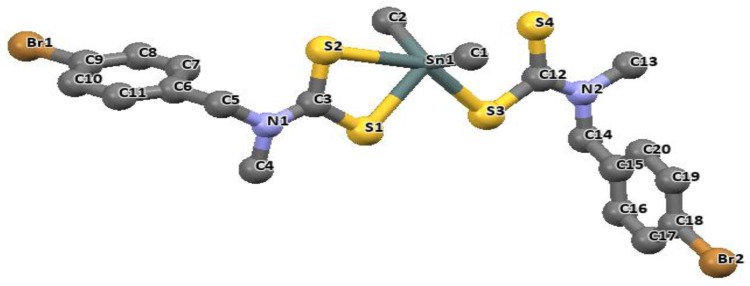
The molecular structure of dimethyltin(IV) bis(*p*-bromo-*N*-methylbenzylamine dithiocarbamate). (For clarity, H atoms were omitted). Redrawn from [83], with permission from Elsevier (Copyright 2018).

**Figure 7:**
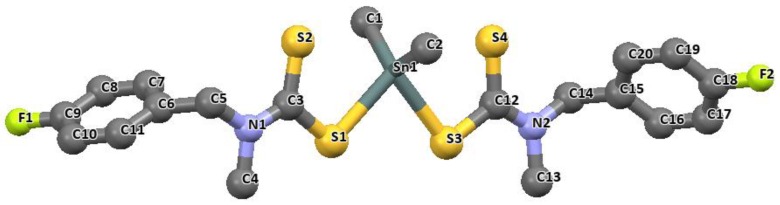
The molecular structure of dimethyltin(IV) bis(*p*-fluoro-*N*-methylbenzylamine dithiocarbamate). (For clarity, H atoms were omitted) [83]. Redrawn from [83], with permission from Elsevier (Copyright 2018).

Some di-organotin(IV) salts have shown, from the crystallographic data obtained, the ability to bind in a monodentate fashion with dithiocarbamate ligand [83,84]. This is uncommon for most substituted di-organotin(IV) complexes which are often found to bond in a bidentate fashion. An example is the di-organotin(IV) complexes of *p*-bromo-*N*-methylbenzylaminedithiocarbamate and *p*-fluoro-*N*-methylbenzylaminedithiocarbamate. The structural data showed that the ligands are bonded in a monodentate fashion. In dimethyltin(IV) bis(-bromo-*N*-methylbenzylamine dithiocarbamate) complex (Figure 6), the bond distances of Sn1-S1 and Sn1-S2 were 2.530(2) Å and 2.515(19) Å respectively, and Sn1–S1 and Sn1–S3 for dimethyltin(IV) bis(*p*-fluoro-*N*-methylbenzylaminedithiocarbamate) were 2.5139(7) Å and 2.5181(7) Å, respectively (Figure 7). The dithiocarbamate ligands in dimethyltin(IV) complexes have similar structural motifs and bonds in similar fashion. The distance of the non-bonded sulfur atoms (S2 and S4) to the Sn atom in dimethyltin(IV) bis(*p*-bromo-*N*-methylbenzylamine dithiocarbamate) are = 2.902(7) and 3.104(9) Å and in dimethyltin(IV) bis(*p*-fluoro-*N*-methylbenzylamine dithiocarbamate) are 2.997(7) and 3.023(8) Å respectively. They are relatively longer than the coordinated Sn–S distances, but significantly shorter than the sum of the van der Waal force. This weak interaction which exists between the S2 and S4 atoms with the tin metal forces the opening of the angle between the C-Sn-C, and the closing up of the angle between the S-Sn-S to a small value, which is less than the expected tetrahedral value of 109°. Hence, resulted into a distortion away from the expected tetrahedral geometry around the Sn center [83].

**Figure 8:**
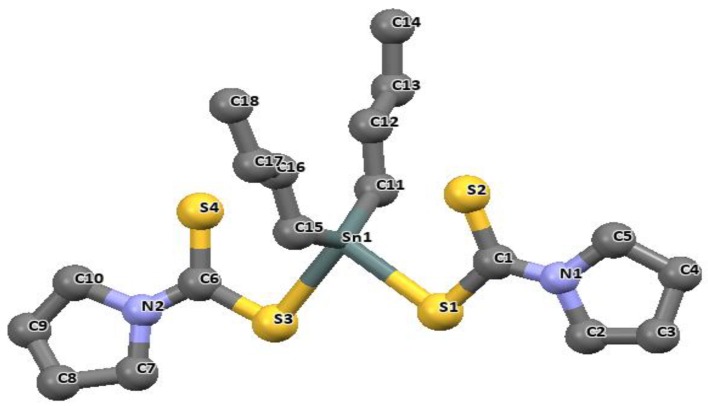
The molecular structure of dibutyltin(IV) pyrrolidinedithiocarbamate complex [Sn{S_2_CN(CH_2_)_4_}_2_n-Bu_2_] and the atom numbering (For clarity, H atoms were omitted). Redrawn from [84], with permission from Elsevier (Copyright 2018).

A distorted tetrahedral geometry has also been reported for the organotin(IV) complex obtained from ammonium pyrrolidinedithiocarbamate ([Sn{S_2_CN(CH_2_)_4_}_2_n-Bu_2_]) [84]. The dithiocarbamate ligand was bonded to the adjacent side of the organotin(IV) moiety via a single sulfur atom at both end as shown in Figure 8. The bond distances obtained for the Sn-S bond are Sn–S (1) = 2.534 Å and Sn–S (3) = 2.532 Å.

**Figure 9:**
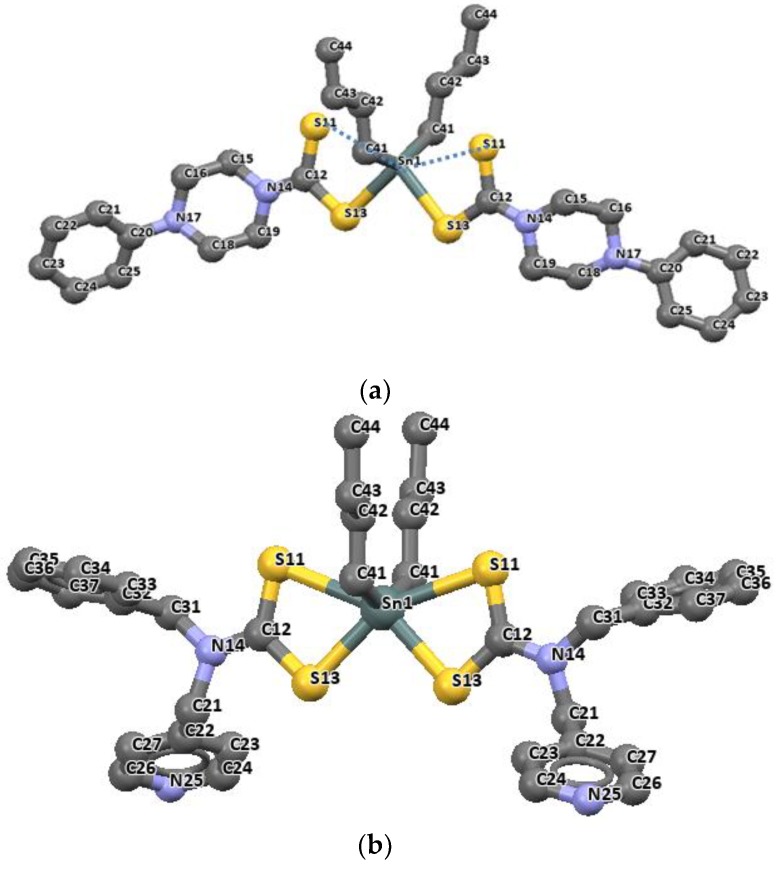

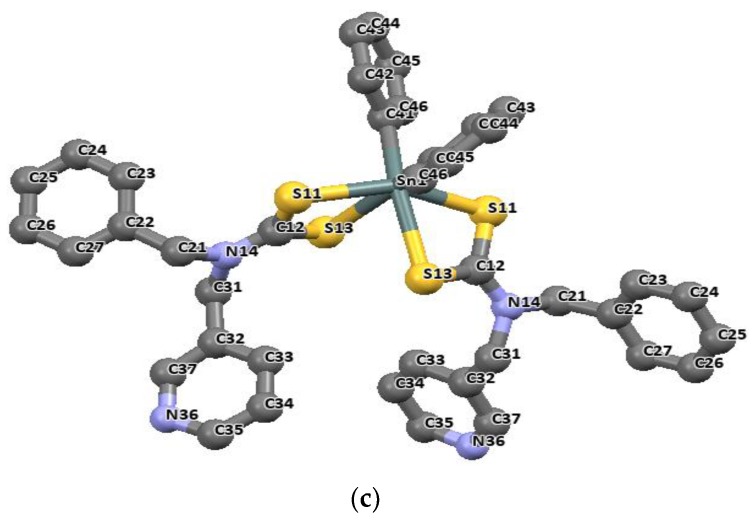
Molecular structures of dibutyltin (IV) 4-phenylpiperazine-1-dithiocarbamate (**a**) [(nBu)_2_Sn(L^1^)_2_], dibutyltin(IV) *N*-benzyl-*N*-methyl-4-pyridyldithiocarbamate (**b**) [(nBu)_2_Sn(L^2^)_2_] and diphenyltin(IV) *N*-benzyl-*N*-methyl-3-pyridyldithiocarbamate (**c**) [(Ph)_2_Sn(L^3^)_2_], the atom numbering scheme (all hydrogen atoms are omitted for clarity). Redrawn from [26], with permission from Elsevier (Copyright 2018).

When a dithiocarbamate ligand bonds with its two sulfur atoms in a bidentate fashion, it often results in an octahedral geometry around the tin atom [85]. This class of organotin(IV) dithiocarbamate complexes is represented in Figure 9a–c. Phenylpiperazine-1-dithiocarbamate [(nBu)_2_Sn(L^1^)_2_] and *N*-benzyl-*N*-methyl-4-pyridyldithiocarbamate [(nBu)_2_Sn(L^2^)_2_], as shown in Figure 9a,b, show that the metal occupies a twofold axis such that half of the molecule is found in the asymmetric unit. The molecular structure and structural data obtained for the dibutyltin(IV) complexes of 4- In the dibutyltin(IV) complexes, the two dithiocarbamate ligands bonds through the two available sulfur (S11 and S13) in an unequal distance (2.533(1)–2.554(1) Å) to the tin atom and at a somewhat longer distance of 2.896(1)–2.971(1) Å respectively.

**Figure 10:**
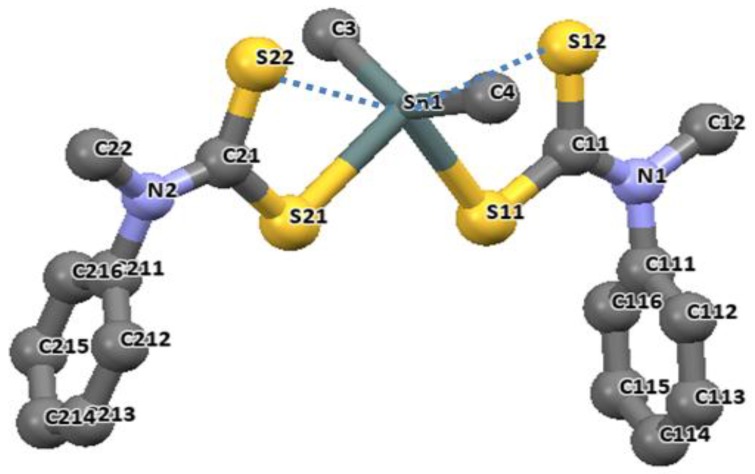
Molecular structure of dimethyltin(IV) complexes of *N*-methyl-*N*-phenyldithiocarbamate [(CH_3_)_2_SnL_2_]. (For clarity, H atoms were omitted). Redrawn from [86], with permission from Elsevier (Copyright 2018).

**Figure 11:**
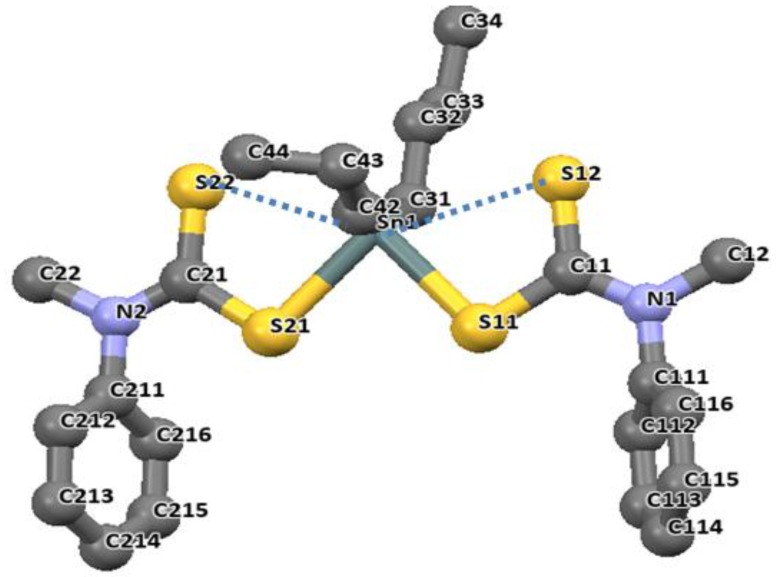
Molecular structure of dibutyltin(IV) complexes of *N*-methyl-*N*-phenyldithiocarbamate [(C_4_H_9_)_2_SnL_2_]. (For clarity, H atoms were omitted). Redrawn from [86], with permission from Elsevier (Copyright 2018).

The longer distances are well within the sum of the van der Waals radii of tin and sulfur (3.97 Å), and are considered as bonds. The distances of Sn-C bonds in these complexes observed in the range 2.140(2)–2.145(5) Å are within the range found for di-organotin(IV) dithiocarbamates. For diphenyltin(IV) *N*-benzyl-*N*-methyl-3-pyridyl dithiocarbamate [(Ph)_2_Sn(L^3^)_2_], similar unequal distances were observed as for Sn-S bond, 2.593(1) and 2.680(1) Å. The Sn-C41 bond length is 2.139(4) Å. the geometry around these complexes is best described as distorted octahedral geometry [26]. Similar geometries involving organotin(IV) complexes of *N*-methyl-*N*-phenyldithiocarbamate of the formular R_2_SnL_2_ (R = CH_3_ and Bu = C_4_H_9_) presented in Figure 10 and Figure 11 have been reported by our research group [86]. The four sulfur atoms of the ligand in complexes [(CH_3_)_2_SnL_2_] and (C_4_H_9_)2SnL_2_ (L = *N*-methyl-*N*-phenyldithiocarbamate) bonded to the central Sn atom in an anisobidentate mode due to the minute opening of the bond angle of C-Sn-C towards 180°; (142.06° for [(CH_3_)_2_SnL_2_] and (137.7° for [(C_4_H_9_)_2_SnL_2_] moving away from 109°. The tin-sulfide bonds in [(CH_3_)_2_SnL_2_] were observed to bond in an unequal distance at both end of the complex. The Sn1-S12 [2.9785(6) Å] and Sn1-S22 [2.9479(7) Å] bonds were longer than the Sn1-S11 [2.5199(7) Å] and Sn1-S21 [2.5259(5) Å]. The Sn-S bond distances observed for [(C_4_H_9_)_2_SnL_2_] similarly show unequal distances for Sn1-S11 [2.5179(8) Å], Sn1-S12 [3.0275(8) Å], Sn1-S21 [2.507(7) Å] and Sn1-S22 [3.0465(9) Å]. Thus, this consequently resulted in an asymmetric molecule [81]. The shorter bond distances have a stronger Sn-S bonds with an acute angle cis to each other at 83.80(2)° while the weaker and the longer Sn-S bond has a bond angle of 146.16(2)° which is less co-linear with each other for [(CH_3_)_2_SnL_2_] [37]. Similar observation was made in [(C_4_H_9_)_2_SnL_2_]. In both complexes, the longer bond distances were found well below the expected van der Wall radii (3.97 Å) [81]. Thus, they were reported as Sn‒S bond, although weaker than a typical Sn-S bonds (2.42 Å). The degree of coordination about the Sn center in this complex resulted in a shift from the expected bite angle of the chelating dithiocarbamate ligand and thus gave a different bond length [81]. The six coordination geometry expected were badly distorted around the central metal due to steric and electronic reasons, and the asymmetric nature of the dithiocarbamate ligands. Therefore, the geometry about the tin center for both complexes is best described as a skew trapezoidal-bipyramidal geometry similar to what has been reported for di-organotin(IV) complexes [87,88].

Contrary to most known structures of di-organotin(IV) complexes of dithiocarbamate, another structural motif exists in which one of the two dithiocarbamate ligands can bond to the central metal atom in a monodentate fashion and the other in a bidentate fashion (with second sulfur hanging as a pendant). This consequently results in five-coordinate geometry around the central Sn atom. An example is the dimethyltin(IV) complex of the *N*,*N*-dimethyldithiocarbamate ligand [79], in which one of the ligands is bonded in an anisobidentate fashion due to the unequal distances of the tin to sulfur bond (Sn-S1 = 2.795(1) and Sn-S3 = 2.489(1) Å) and a bite angle of 67.56(2)°. The non-coordinating pendant sulfur atom S4 possesses a distance of 3.532(1) Å. The geometry around the tin metal observed has been described as a distorted trigonal bipyramid. The two bulky groups of the tert-butyl ligand moiety has been suggested to be responsible for the five coordinate geometry around the Sn center [79].

**Figure 12:**
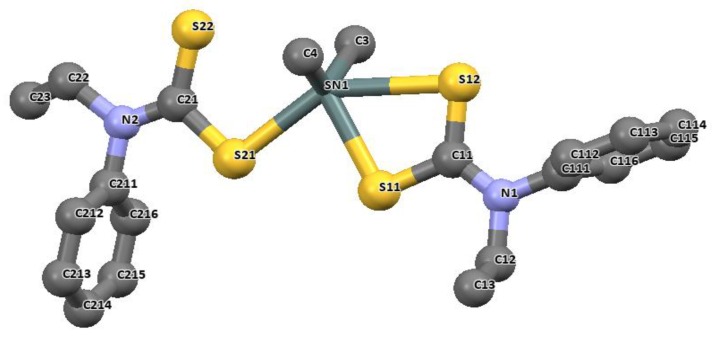
The molecular structure of dimethyltin(IV) complexes of *N*-ethyl-*N*-phenyl dithiocarbamate [Me_2_SnL_2_]. (For clarity, H atoms were omitted). Redrawn from [89], with permission from Elsevier (Copyright 2018).

**Figure 13:**
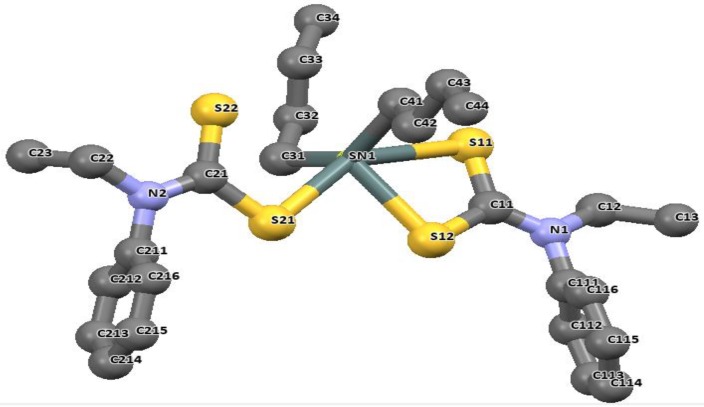
The molecular structure of dibutyltin(IV) complexes of *N*-ethyl-*N*-phenyldithiocarbamate [Bu_2_SnL_2_]. (For clarity, H atoms were omitted). Redrawn from [89], with permission from Elsevier (Copyright 2018).

We have reported similar geometry involving dimethyltin(IV) and dibutyltin(IV) complexes of *N*-ethyl-*N*-phenyldithiocarbamate represented as [Me_2_SnL_2_] and [Bu_2_SnL_2_] respectively (Me = methyl, Bu = butyl and L = *N*-ethyl-*N*-phenyldithiocarbamate). The structures are presented in Figure 12 and Figure 13 [89]. The bidentate dithiocarbamate ligands in both complexes are bonded in an anisobidentate fashion to the Sn atom due to the unequal bond distances of the Sn to S in complex [Me_2_SnL_2_]: Sn1-S11 [2.5193(5) Å], Sn1-S12 [2.9135(6) Å]) and a bite angle of [65.50(2)°], and in [Bu_2_SnL_2_]: Sn1-S12 [2.5250(7) Å], Sn1-S11 [2.8619(8) Å] with a bite angle of [66.39(2)°]. The adjacent dithiocarbamate bonds to the tin atom via a single sulfur atom in a mondentate fashion in both complexes with bond distances of 2.5193(5) and 2.5318(8) Å in [Me_2_SnL_2_] and [Bu_2_SnL_2_] respectively. A non-bonding distance of the pendant sulfur to the metal atom (S22 atom at 3.12(6) and 3.12(9) Å) has been observed in each of the complexes. This was ascribed to a weak coordination of a sulfur atom due to a change in one of the short Sn-S bonds in the dithiocarbamate moiety, consequently leading to a positional change which is approximately orthogonal to the Sn-S22 plane [15].

The geometry around the Sn center was best described as trigonal bipyramidal. This due to the equatorial position occupied by the alkyl groups on the organotin(IV) moiety and the axial position occupied by the sulfur atoms [S12-Sn1-S21] 144.91(2)° in the dimethyltin(IV), and [S11-Sn1-S21] 147.88(3)° in the dibutyltin(IV) complexes. The percentage along the trigonal bipyramid to square pyramid berry pseudorotation was 18.8 and 16.7 for [Me_2_SnL_2_] and [Bu_2_SnL_2_] respectively.

### Thermal Decomposition Studies

Thermogravimetric analyses of compounds are usually carried out in order to gain insight into the thermal properties and stability of the compounds; and in most dithiocarbamate complexes it helps ascertain the possible use of the complexes as single source precursors for metal sulfides nanoparticles. In the case of organotin(IV) dithiocarbamate, a possible formation of tin sulfide is envisaged [81]. The thermochemistry of metal dithiocarbamates has shown, from its wide study, that the complexes either volatilize to leave a negligible amount of residue or decompose to yield metal sulfide [90].

The major techniques employed for the thermal study of a wide variety of metal dithiocarbamate complexes are TGA, STA and DSC [91]. These techniques have made it possible to study the trend within a group of complexes with relevant analytical and structural significance, and also classify them based on their thermal properties. A common intermediate that is associated with the thermal decomposition of metal dithiocarbamate is metal thiocyanate intermediate [92]. Metal thiocyanate has been reported as an intermediate for a number of transition metal dithiocarbamate complexes which often decomposes to give their corresponding metal sulfides as final residues [93,94,95,96]. The decomposition pattern of some organotin(IV) dithiocarbamate complexes can also progress via the tin thiocyanate intermediate to give a tin sulfide residue [86]. Thermal study data relating to the decomposition of tin dithiocarbamates are available [91], but it has been observed (generally) that there is no clear cut trend in the thermal decomposition pattern within this group of organotin(IV) complexes irrespective of structural similarity. Tin dithiocarbamates exhibit complicated thermochemistry and an unpredictable thermal decomposition pattern even for a series of structurally related complexes [91]. Even among structurally related compounds, it is almost impossible to establish a trend because they do not follow similar decomposition pathway [91].

Two types of residues, including tin sulfide or tin oxide, are often obtained from the thermal decomposition of organotin(IV) dithiocarbamate complexes, and they are dependent on the decomposition condition (inert or in air) [97]. Tin sulfides can exist in three possible phases: SnS that exhibit layer structures, SnS_2_ and Sn_2_S_3_ which has a mixed valence of Sn^2+^/Sn^4+^ with a ribbon-like structure [16]. Tin sulfide has semiconducting properties; and due to its low toxicity, it is used as a solar energy absorber in holographic recording and for infrared detection [98]. Tin sulfide in its various forms possesses the following band gaps: SnS (1.3 eV), SnS_2_ (2.18 eV), and Sn_2_S_3_ (0.95 eV). The electronic band gap of SnS has attracted much attention because it lies between that of Ga and Si [98].

Tin sulfide nanoparticles of different morphologies such as rods, belts, and spheres have been produced via the thermal decomposition of organotin(IV) dithiocarbamate complexes under specific conditions [99]. Hence, it is imperative to understand the thermal behavior of organotin(IV) dithiocarbamate complexes [91]. New routes for making cheaper and micro scale inorganic material with well-defined sizes and shapes have led to the continuous research in materials science. Dithiocarbamate complexes, on the other hand, have been regarded as a good single source materials for metal sulfides [100]. This is because they possess sulfur as the only heteroatom to the metal center (after their thermal decomposition) while avoiding the formation of undesired impurities like metal oxides to exclusively yield metal sulfide nanoparticles [101]. The product of the thermal decomposition of organotin(IV) dithiocarbamate has various applications. For example, tin oxides are used as light detectors (visible and infrared), solar cells, light emitting diodes and as gas sensors [97]. Tin sulfides have outstanding electronic and optical properties due to their narrow bad gap. They have been used to replace cadmium chalcogenides (due to toxicity) or silicon based sensors in low cost green photovoltaic devices [16].

Thermogravimetric analyses have been carried out (under nitrogen) on some organotin(IV) 4-hydroxypiperidine dithiocarbamate. The decomposition of these complexes proceeded via a single step decomposition regardless of the nature of the alkyl group attached. The final residue obtained for these sets of complexes was found to be SnS [81]. Similarly, the TGA of (diphenyltin(IV) *N*-benzyl-*N*-methyl-3-pyridyldithiocarbamate and dibutyltin-4-ethoxycarbonyl piperidine-1- dithiocarbamate) at similar heating rate of 10 °C min^−1^ (to a temperature of 600 °C) under nitrogen gave a double decomposition pattern leaving SnS_2_ and SnS residue respectively [26].

**Figure 14:**
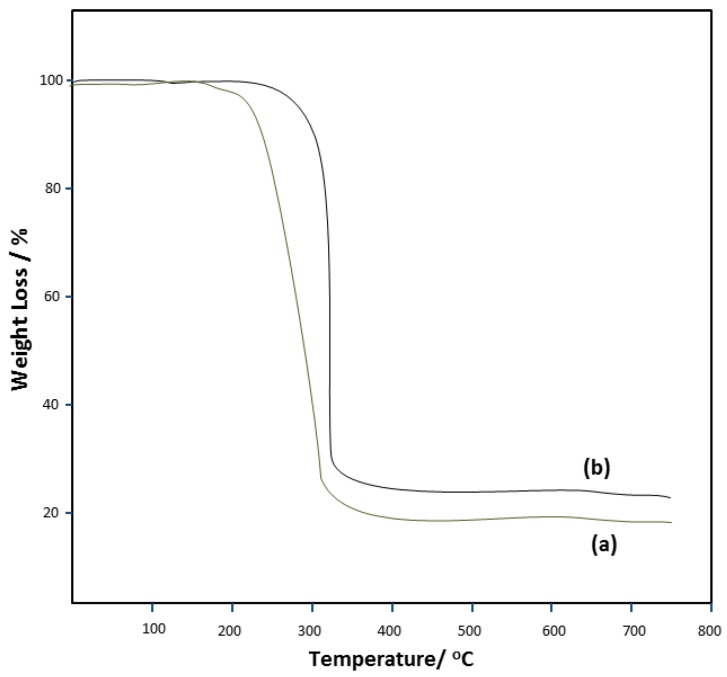
Thermogravimetric curves of (a) [Sn{S_2_CN(CH_2_)_4_}_2_{C_6_H_5_}_2_] and (b) [Sn{S_2_CN(CH_2_)_4_}{C_6_H_5_}_3_] in N_2_ [16]. Redrawn from [16], with permission from Elsevier (Copyright 2018).

In the report of Menezes et al., [16], the thermal study of two organotin(IV) complexes of ammonium pyrrolidinedithiocarbamate with the formula [Sn{S_2_CN(CH_2_)_4_}_2_{C_6_H_5_}_2_] and [Sn{S_2_CN(CH_2_)_4_}{C_6_H_5_}_3_ ] were carried out. The decomposition of these complexes were observed to proceed in a pseudo-single step which began at 207 and 233 °C (±2 °C) for [Sn{S_2_CN(CH_2_)_4_}_2_{C_6_H_5_}_2_] and [Sn{S_2_CN(CH_2_)_4_}{C_6_H_5_}_3_] respectively, and terminates at about 350 °C in both cases as shown in Figure 14. In [Sn{S_2_CN(CH_2_)_4_}{C_6_H_5_}_3_], it was observed that the presence of an extra phenyl group increased temperature of decomposition by approximately 26 °C. The final residues of these decompositions were chemically and structurally characterized. The electron probe micro analyzer (EPMA) results, clearly shows the presence of S and Sn as the major elements found in the residues. However, a minute amount of O_2_ was observed and attributed to the formation of SnO_2_ as a much lesser product. This O_2_ was suggested to originate from the N_2_, which was not completely free from moisture or oxygen. The pattern observed from the diffraction of these residues suggested the formation of a mixed phase γ-Sn_2_S_3_ and SnS for both orthorhombic phases, in view of the diffraction lines.

**Figure 15:**
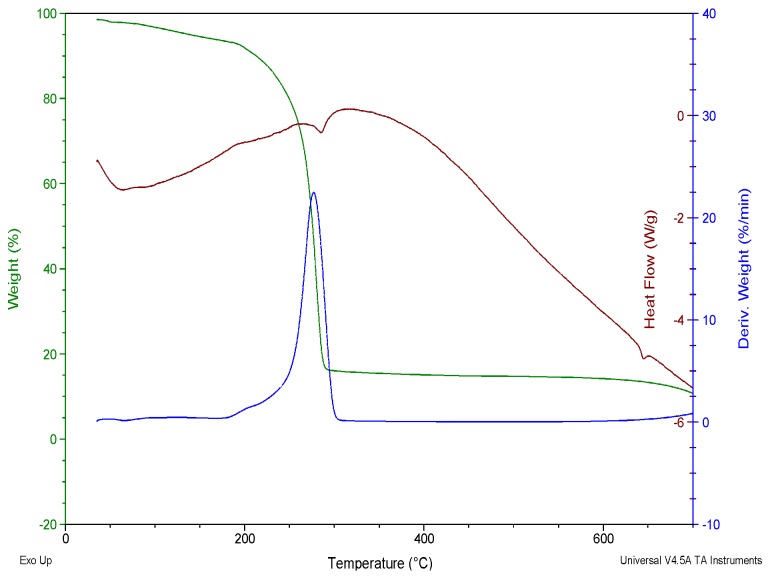
TG/DTG and DSC curves of diphenyltin(IV) *N*-ethyl-*N*-phenyl dithiocarbamateobtained in N_2_ (75 mL/min at heating rate 10 °C/min). copied from [89] with permission from Elsevier (Copyright 2018).

**Figure 16:**
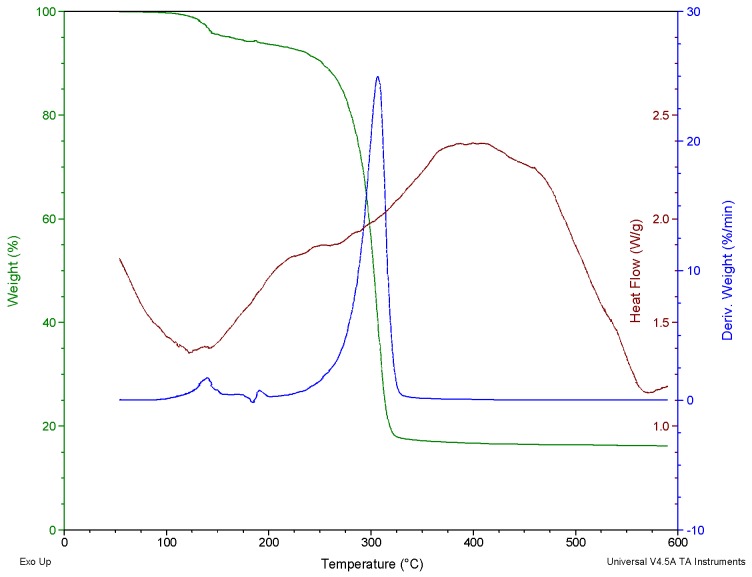
TG/DTG and DSC curves of diphenyltin(IV) *N*-methyl-*N*-phenyl dithiocarbamate obtained in N_2_ (75 mL/min at heating rate 10 °C/min. Copied from [86] with permission from Elsevier (Copyright 2018).

Our group has also reported the thermal decomposition study of some mono and di-organotin(IV) complexes of some *N*-alkyl-*N*-phenyldithiocarbamate complexes [86,89]. The thermal properties between 50 to 700 °C of the complexes were studied in an inert environment. Few of the complexes decomposed via tin thiocyanate intermediate but no noticeable trend was observed in the decomposition pattern of these complexes. Although these complexes are structurally related, different decomposition patterns/steps and intermediates were observed for each of the complexes as shown in Figure 15 and Figure 16. However, the complexes gave only SnS as the final residue with no additional phase/product.

## 3. Biological Applications of Organotin(IV) Dithiocarbamate

### 3.1. Antimicrobial Properties of Organotin(IV) Dithiocarbamate

The various antimicrobial activities of organotin(IV) dithiocarbamate complexes originate from the individual properties associated with organotin(IV) and dithiocarbamates. Generally, the antimicrobial mechanisms of action involve interference with the cell wall synthesis (by altering the cell permeability), metabolic interference with various cellular enzymes, cellular impairment due to denaturing of proteins, and alteration of the normal cell processes due to the hydrogen bonding through the azomethine group with active cellular constituents [102]. The stereo-electronic properties of sulfur containing ligands have been attributed to their good activity as antimicrobial agents which is enhanced upon coordination to metals [17], while the antimicrobial activities of organotin(IV) complexes are often governed by their molecular weight and lipophilicity [39]. Complex formation causes an increased antimicrobial activity [103], which, in turn, alters the polar nature of the metal by the delocalization of electron and charge sharing with sulfur donor groups. This electron delocalization enhances plasma membrane permeability for the complexes [14]. Rehman et al., have suggested the mechanism of complexes with organotin(IV) moiety within their structure to proceed either by inhibiting the active site for multiplication or by killing of the microorganism [104]. Gram positive bacteria are known to have a simpler cell wall compared to the more complex wall of the Gram negative bacteria, thus, are more induced by the complexes to give higher activity. The complexity observed in the Gram negative bacteria has been attributed to the outer-lipid membrane formed from lipopolysaccharide contributing to the antigenic specificity of the Gram negative bacteria [38].

#### 3.1.1. Antibacterial Studies

Organotin(IV) dithiocarbamate complexes, due to their useful biological potentials, have been utilized as important pharmacophore in the development of metal based drugs [86]. Organotin(IV) compounds as well as dithiocarbamate compounds, have been used in their uncombined state to develop several antimicrobial agents [32,33,84]. The potential activities of these compounds on individual basis, therefore indicate that both compounds can be combined to give a hybrid molecule with enhanced biological properties which could exert some synergistic biological activity [84,105]. Some currently used antibacterial drugs are known to contain metal fragments. These central metal ions help in maintaining proper structure and/or enhance their activities as antibiotics. Removal of the metal ions from these antibiotics could result into transformation and loss of activity [106]. Among this group of antibiotics with metal centers are bleomycin, streptonigrin, and bacitracin [106]. Drug resistance is a serious global challenge and remains the greatest threat to prevention and treatment of infectious diseases. According to WHO, antimicrobial resistance is the opposition of a microorganism towards the drug that effectively treats the infection caused by the pathogen. Despite the various research and usage of antibiotics, most of them are often accompanied by the evolution of microbial resistance [107]. Hence, the need for continuous search for alternative drugs that can effectively combat pathogenic organism.

Awang et al., synthesized some organotin(IV) dithiocarbamate compounds with the molecular formula R_m_Sn[S_2_CN(CH_3_)(C_6_H_11_)]_4-m_ bearing different alkyl and phenyl derivatives (where m = 2, R = CH_3_, C_2_H_5_; m = 3, R = C_6_H_5_) which were screened against four bacteria: *Staphylococcus aureus*, *Salmonella typhimurium*, *Pseudomonas aeruginosa* and *Bacillus subtilis*. The studied complexes generally gave moderate to good antibacterial activity. It was noted that the phenyl containing complex showed inhibitory effect on selected bacteria strains. Cytotoxicity assays of the compounds confirm the potential of these complexes for further research [24]. Furthermore, they synthesized some organotin(IV) complexes derived from methyltin(IV), butyltin(IV), phenyltin(IV), methyltin(IV), butyltin(IV), phenyltin(IV) and the ligands methylcyclohexyldithiocarbamate and ethylcyclohexyldithiocarbamate. These complexes were also screened for their antimicrobial properties against the ESKAPE bacteria, i.e., *Enterococcus raffinosus*, *Staphylococcus aureus*, *Klebsiella sp.*, *Acinetobacter baumanni*, *Pseudomonas aeruginosa*, and *Enterobacter aerogenes,* using disc diffusion and microdilution methods. It was observed that phenyltin(IV) methylcyclohexyldithiocarbamate derivative showed good activity towards most bacteria tested similar to ampicillin (2.5 mg/mL) in the minimum inhibitory concentration (MIC). Overall, all the complexes showed bacteriostatic effects at the minimum bactericidal concentration (MBC) higher than the MIC values, but phenyltin(IV) complex exhibited the best activity and has potential as an antibacterial agent upon further clinical investigation [108].

Good activities have been reported by Sainorudin et al., [53] for dibutyltin(IV) and triphenyltin(IV) complexes of di-2-ethylhexyldithiocarbamate and *N*-methylbutyldithiocarbamate ligands. These complexes were screened against some bacterial strains including *Escherichia coli*, *Staphylococcus aureus*, *Salmonella typhi* and *Bacillus cereus* using penicillin and streptomycin as positive and negative control respectively. These complexes showed good antimicrobial activity and, thus, can be useful as anitibacterial agents [53]. Similarly, Adli et al., screened some organotin(IV) dithiocarbamate complexes of 1-methylpiperazinedithiocarbamate and *N*-methylcyclohexyl-dithiocarbamate against *Staphylococcus aureus*, *Bacillus cereus*, *Salmonella typhi* and *Escherichia coli*. The microbial test carried out showed all synthesized complexes possessed great potential as antimicrobial agents [65].

Some aforementioned organotin(IV) complexes of *N*-methyl-*N*-phenyldithiocarbamate synthesized by our research group have been screened *in vitro* against some bacterial organisms (*S. Aureus, B. cerues, K. pneumonia, P. aeruginosa* and *E. coli*) for their antimicrobial potentials. It was observed that the phenyl and diphenyltin complexes showed the best antibacterial activity (within the series) with inhibition diameter ranging between 10 to 21 mm. This was attributed to the presence a planar phenyl groups attached to the tin center which increases the lipophilic properties of the complexes through the lipid bilayer of the bacteria organisms. Generally it was observed that the complexes showed good activity against gram negative bacterial activity due to their lack of cell wall. The Gram positive organism on the other hand possess a complex cell wall due to an outer-lipid membrane formed from lipopolysaccharide contributing to the antigenic specificity and, therefore, prevents the permeation of the complexes into these organism. The complexes, when compared to a standard antibacterial drug, showed a moderate activity and thus, could be an alternative antibacterial drug after some clinical trials [86].

#### 3.1.2. Antifungal Studies

Although resistance to antibiotics by bacteria has become a widely recognized public health threat, not much is known about the effects of drug-resistance in fungal infections. Just as there has been increase in the discovery and usage of antibacterial drugs with increase in high-risk patients, discoveries of the widespread and increasing rates of serious invasive fungal infections have also been recorded and the available antifungals have become less effective against certain infections due to emergence of drug-resistance [109]. There has been an increase in the number of deaths caused by *Aspegillosis* (a variety of diseases caused by fungi infection of the genus *Aspergillus*) which has been attributed to the action of immunosuppressive drugs, diseases, cancer or AIDs. In the treatment of aggressive aspergillosis, liposomal amphotericin B and voriconazole are the first choice drugs which can be dangerous to the kidney and liver. Resistance to antifungal drugs in conventional treatment has been found to be related to target alterations of the drug, ergosterol biosynthesis interference and/or increase in the expression of drug efflux pumps. These reasons have made the need for alternative drugs very important and synthesis of metal-based drugs might provide a novel platform for new alternative antifungal agents, either alone or in combination with already used drugs to overcome resistance [110]. Tri-oganotin(IV) complexes [SnPh_3_{S_2_CNR(R^1^)}], [SnCy_3_{S_2_CNR(R^1^)}], [SnMe_3_{S_2_CNR(R^2^)}], [SnPh_3_{S_2_CNR(R^2^)}] and [SnCy_3_{S_2_CNR(R^2^)}], [R = CH_3_, R^1^ = CH_2_ CH(OMe)_2_, and R^2^ = 2-methyl-1,3-dioxolane] have been isolated, and screened by Ferreira et al., against *Aspergillus flavus, Aspergillus niger, Aspergillus parasiticus* and *Penicillium citrinum*. Good antifungal activities were observed which was in correlation with a study on the structural-activity relationship (SAR) carried out. Complex [SnPh_3_{S_2_CNR(R^2^)}] showed a noteworthy biocidal activity. Complexes [SnPh_3_{S_2_CNR(R^1^)}] and [SnPh_3_{S_2_CNR(R^2^)}] exhibited a nanomolar inhibition concentration in terms of IC_50_ [111]. Some tin(IV) complexes of pyrrolidinedithiocarbamate, [Sn{S_2_CN(CH_2_)_4_}_2_Cl_2_], [Sn{S_2_CN(CH_2_)_4_}_2_Ph_2_], [Sn{S_2_CN(CH_2_)_4_}Ph_3_], [Sn{S_2_CN(CH_2_)_4_}_2_n-Bu_2_], [Sn{S_2_CN(CH_2_)_4_}Cy_3_] have been screened for antifungal activities using agar disk diffusion against human pathogenic fungi (*Candida albican*). The complexes were active with inhibition zones ≥ 11 mm, intermediate inhibition zones within the range 11–9 mm and resistant ≤ 9 mm. The inhibition zone for the commercial nystatin was 14 mm and was used to compare the activities of the complexes. The *C. albicans* exhibited resistance against pyrrolidinedithiocarbamate ligand but showed moderate activities with most of the tin(IV) complexes. Complexes [Sn{S_2_CN(CH_2_)_4_}_2_Cl_2_] and [Sn{S_2_CN(CH_2_)_4_}_2_n-Bu_2_] showed the highest activities. The penetration of the complexes through the yeast membrane was attributed to the size and structure of the complexes. The two chlorine atoms in complex [Sn{S_2_CN(CH_2_)_4_}_2_Cl_2_] were attributed to its ease of penetration across the cell membrane compared to other complexes [84]. Organotin(IV) methyl- and ethylisopropyldithiocarbamate complexes: dimethyltin(IV) methyl-isopropyldithiocarbamate, dibutyltin(IV) methylisopropyldithiocarbamate, triphenyltin(IV) methylisopropyldithiocarbamate, dimethyltin(IV) ethylisopropyldithiocarbamate, dibutyltin(IV) ethylisopropyldithiocarbamate, triphenyltin(IV) ethylisopropyldithiocarbamate have been synthesized and their biological activities were evaluated. The antifungal activities of these complexes were screened against three species of fungi namely *Aspergillus niger, Candida albicans,* and *Saccharomyces cerevisiae* using qualitative disc diffusion and quantitative broth microdilution method. Very good antifungal activities were observed for all the fungi tested. Minimum inhibitory concentration (MIC) values obtained for these compounds were better than streptomycin against *B. cereus*, *S. aureus*, and *S. mutans* [18].

### 3.2. Anticancer/Antitumor Studies

Metal complexes have, overtime, proven to be useful in the fight against cancer. Cisplatin, an inorganic complex of platinum discovered by Rosenberge, exhibits antitumor characteristics, and remains one of the world’s bestselling anticancer drugs. This discovery led to changes in the course of therapeutic management of several tumors, such as those of the ovary, testes, head and neck [112]. However, this spectacular clinical activity of cisplatin has been found to be limited by significant side effects such as DNA damage and the emergence of drug resistance [113]. As a result of this usual accompanied side effect of the platinum based antitumor drug, further research for alternative metal based drugs with improved antitumor activities such as the non-platinum based anticancer complex have been initiated. Good activities have been recorded with metals such as copper, gold [114], gallium, germanium, tin, ruthenium, iridium, lanthanum [115], but none has entered the clinical trial stage till date. Among the afore mentioned, gold and tin derivatives have gained increased interest and have appeared very promising as potential anticancer drugs [116]. Hence, the continual search for novel chemotherapeutic agents with decreased toxic side-effects and improved specificity. In vitro screening of new coordination complexes, followed by careful selection of best active anticancer compounds, remains the best way of identifying potential antitumor agents. The underlying mechanism of the organotin(IV) compounds involve the inhibition of F1F0 ATP synthase [117,118]. F1F0 ATP synthase have been found to be harmful to all life forms [118]. In their uncombined form as ordinary salts, organotin(IV) compounds such as tributyltin(IV) chloride have been reported to inhibit ATP synthases by targeting the Na^+^ channel. Likewise, oxidative phosphorylation and ATP synthesis in the mitochondria has been suggested to be suppressed by the dibutyltin(IV) dichloride [119]. Other reports have suggested their mechanism of action to involve binding to both DNA base and phosphate contrary to the cisplatin which could bind to DNA with a cross link. This can induce DNA damage by inhibiting DNA and protein synthesis while increasing RNA synthesis, thereby affecting structural selectively [120]. These observed modes of actions have been suggested to hinder the usage of organotin(IV) compounds as improved antitumor agents due to the cellular targets of these compounds in their mechanism regardless of the mitochondrial oxidative phosphorylation [6]. Nevertheless, there is a need to further understand how to modulate these toxic effects.

Various studies with interesting results have been conducted by Amir et al., on the in vitro antitumor properties of organotin(IV) complexes of dithiocarbamate against a wide panel of tumor cell lines of human origin [121]. Series of phenyltin(IV) dithiocarbamates of the formula Ph_4-n_Sn(S_2_CNEt_2_)_n_, where n = 1–3, were evaluated for their cytotoxicity against L1210 mouse leukaemia cell line. About 50% growth inhibition rating of 0.3 µM was found compared with 0.6 and 1.2 µM for the drugs cisplatin and carboplatin respectively. It was also discovered that when chloride was introduced, as in PhSn(S_2_CNEt_2_)_2_Cl and Ph_2_Sn(S_2_CNEt_2_)Cl, it resulted in low toxicity. Nevertheless, it was found to still exert more cytotoxic effect than drugs containing Pt metal [15]. In another study, organotin(IV) dithiocarbamates with general formula (ArCH_2_)_2_Sn(S_2_CNR’_2_)Cl and (ArCH_2_)_2_Sn(S_2_CNR’_2_)_2_ were evaluated against various panels of human cancer cell lines. It was reported that the group with chloride were more cytotoxic than their counterparts without chloride substituents, i.e., (ArCH_2_)_2_Sn(S_2_CNR’_2_)_2_. From this study it was suggested that the ease of hydrolysis of the former might be the reason for such behavior. The complex, (4-NC-C_6_H_4_CH_2_)_2_Sn[S_2_CN(CH_2_CH_2_)_2_NCH_3_]Cl, was found to be the most cytotoxic compound in the series, and more cytotoxic than cisplatin [63]. Kadu et al., synthesized some binuclear diphenyltin(IV) dithiocabamate macrocyclic complexes through a self-assembly process involving novel diamino precursors 4,4′-bis(2-(alkylamino)acetamido)diphenyl ethers, CS_2_ and Ph_2_SnCl_2_. Using the 3-(4,5-Dimethylthiazol-2-yl)-2,5-diphenyltetrazolium bromide (MTT) assay, cytotoxic activity was carried out against HEP 3B (hepatoma) and IMR 32 (neuroblastoma). The complexes were extremely active against both cell lines and cytotoxicity data having better 16-fold potency compared to cisplatin. The flow cytometric analysis of annexin V–propidium iodide-stained cells of L5, 4 and 6 was found to possess the ability to induce apoptosis in HEP 3B and IMR 32 cells, which is a requirement for major cancer therapy. The binuclear complex shows an extraordinary potency which can be correlated to result from the higher LUMO energy together with the great charge value on the Sn(IV) center [17].

Organotin(IV) compounds of the type R_3_SnCl and R_2_SnCl_2_ (R = C_6_H_5_ or C_4_H_9_) with methoxyethyldithiocarbamate dithiocarbamate ligands have been evaluated for their in vitro anti-proliferative activities against HL-60 cell lines. The results showed that both complexes exhibited high cytotoxicities toward HL-60 cell lines with the IC_50_ values below 1 mM. These complexes were found to possess high anti-proliferative effects; thus, in correlation with an earlier finding of Awang et al., (2010). The potency against the HL-60 cells and the IC_50_ of triphenyltin(IV) complex was much less than the dibutyltin(IV) complex which displayed a higher potential. It was concluded that both complexes have the potential of becoming a good antitumor agents due to their potent cytotoxic effect at micromolar concentrations [57].

### 3.3. Other Biological Studies

The biological applications of organotin(IV) dithiocarbamate are not only limited to the highlighted areas, other biological activities of organotin(IV) dithiocarbamates have also been reported. Organotin(IV) dithiocarbamate have shown good activities as antileishmainial agent. Series of complexes of di- and tri- organotin(IV) dithiocarbamates with 4,4-trimethylenedipiperidine-1-carbodithioate have shown better antileishmanial activity above the currently used standard drug. Their high activities have been attributed to low binding energy with enzyme trypanothione synthetase. It was concluded that, with further clinical investigations the synthesized complexes have a promising potential for the treatment of leishmaniasis [122].

Promising insecticidal activities have been obtained in a study involving complexes of tri-organotin(IV) dithiocarbamates of the formula R_3_SnS_2_CNR_2_′ by Eng et al., (where R = Cy, Ph; NR_2_′ = NEt_2_,N(*n*-Bu)_2_,N(i-Bu)_2_, N(*n*-Pr)_2_,N(CH_2_)_5_, NH(*n*-Pr), NH(*n*-Bu), NH(*i*-Bu)). This was achieved against the second larval instar of the *Anopheles stephensi* and *Aedes aegypti* mosquitoes. These studies showed no significant activities with triphenyltin and tricyclohexyltin derivatives but showed moderate action against the larvae of both species [123].

The scavenging potential for radicals of some organotin(IV) dithiocarbamate complexes of Cyclohexylcarbamodithioic acid have been studied. This was achieved using 2,2-diphenyl-1-picrylhydrazyl (DPPH) radical assay. The IC_50_ values and the percentage inhibition of both the ligand and complexes were obtained and compared with a known antioxidant agent (butylated hydroxytoluene). The ligand itself showed a good radical scavenging potential which was enhanced upon coordination to the respective organotin(IV) salts [38].

### 3.4. Limitation Associated with Toxicity of Organotin(IV) Compound

Several reports have been made about the diverse use of organotin(IV) compounds as biological agents with better activity. Nevertheless, most of these compounds have not been cleared for clinical practice because most derivatives are generally very toxic [6]. They often leave undesirable effects on the nervous, reproductive, endocrine and other systems of animals. Some of which includes cognitive impairment in learning and memory [120]. One factor that increases the toxicity of organotin(IV) compounds is the number of alkyl groups present within the moiety [6]. The bulkiness and the number of these alkyl groups affect the toxicity of the molecule which in turn affects their hydrophilicity at large [124]. Trimethyltin(IV) have been used as stabilizers in PVC, as disinfectants and as insecticides but has a strong neurostatic effect and has been reported to impair spatial memory in rats and mice [120]. Dibutyltin(IV) compounds have also shown neurostatic effect by aggregating on the brain cell cultures.

In platinum chemotherapy, sulfur-containing molecules have been used as chemo-protectants due to their anticancer potential and the ability to modulate cisplatin nephrotoxicity [17]. The mechanism of this modulation is still unknown, but there are reports that propose the DNA to be the probable target of the cytotoxic activity [6,125]. Therefore, regardless of the associated toxicity, organotin(IV) compounds and their derivatives can have their toxicities modulated by a judicious choice of the ligand (like dithiocarbamate) coordinated to the organotin(IV) moiety to minimize this drawbacks [6].

## 4. Conclusions

The chemistry and the synergy created by the individual moieties of organotin(IV) and dithiocarbamate compounds has led to the enhanced biological activity of organotin(IV) dithiocarbamate complexes. The large volume of research data available for these sets of compounds has made it possible to predict the geometry and also consider them for other conceivable applications. A major attribute of these complexes is their ability to have the coordination number around the tin center increased and thus influence the geometry in the complexes. Their geometries are not exactly perfect, but are distorted due to the asymmetric nature of the dithiocarbamate ligand which consequently leads to unequal distances of the tin-sulfur bond. The possibilities for several resonance structures of the dithiocarbamate moiety explain their outstanding stability. Numerous applications of dithiocarbamate compounds stems from the ease of replacing the cations (K^+^/Na^+^/NH_4_^+^) in a coordinate bond through the sulfur atom to form a complex with a metals. Thermal studies of these complexes have shown that they could be useful single source precursors for tin-sulfide nanoparticle which can exist as SnS, SnS_2_, or Sn_2_S_3_ with good semiconducting properties. Hence, these compounds also found relevance in Materials chemistry.

The various biocidal activities of organotin(IV) dithiocarbamate complexes are worthy of development and utilization. Their usage should not only be limited to their application in the industries, agriculture and material science but can be further explored for the treatment of diseases as described and enumerated here. The use of these complexes in drug design could make a significant contribution to human lives, and also provide alternative medication which might be cheaper and better than the currently available drugs. The World Health Organization [126] recently gave a report about the availability of antibiotics and the an alarming encroachment of resistance to the modified existing class of the currently used antibiotics. Hence, the need for new therapeutic agents to combat this menace is imperative. Organtin(IV) dithiocarbamate complexes, therefore, can prove useful in the fight against antimicrobial resistance and against other infectious diseases due to the diverse biological potentials. However, considerable effort must be made to understand their specific mechanism of action and side effects which is still a drawback in their usage. Regardless of the side effects, these complexes can offer a platform for the design of novel therapeutic agents, thus new approaches/ideas and detail studies are needed to outwit these drawbacks.
